# Dose-finding studies in drug development for rare genetic diseases

**DOI:** 10.1186/s13023-022-02298-6

**Published:** 2022-04-05

**Authors:** Lingshan Wang, Jie Wang, Ji Feng, Mary Doi, Salvatore Pepe, Michael Pacanowski, Robert N. Schuck

**Affiliations:** 1 Office of Clinical Pharmacology, Office of Translational Sciences, Center for Drug Evaluation and Research, US Food and Drug Administration, Silver Spring, MD USA; 2 Office of Translational Sciences Immediate Office, Center for Drug Evaluation and Research, US Food and Drug Administration, Silver Spring, MD USA

**Keywords:** Clinical pharmacology, Dose-finding, Biomarker, Drug development, Orphan drug, Rare disease

## Abstract

**Background:**

The small patient populations inherent to rare genetic diseases present many challenges to the traditional drug development paradigm. One major challenge is generating sufficient data in early phase studies to inform dose selection for later phase studies and dose optimization for clinical use of the drug. However, optimizing the benefit-risk profile of drugs through appropriate dose selection during drug development is critical for all drugs, including those being developed to treat rare diseases. Recognizing the challenges of conducting dose finding studies in rare disease populations and the importance of dose selection and optimization for successful drug development, we assessed the dose-finding studies and analyses conducted for drugs recently approved for rare genetic diseases.

**Results:**

Of the 40 marketing applications for new molecular entity (NME) drugs and biologics approved by the United States Food and Drug Administration for rare genetic diseases from 2015 to 2020, 21 (53%) of the development programs conducted at least one dedicated dose-finding study. In addition, the majority of drug development programs conducted clinical studies in healthy subjects and included population pharmacokinetic and exposure–response analyses; some programs also conducted clinical studies in patient populations other than the disease for which the drug was initially approved. The majority of primary endpoints utilized in dedicated dose-finding studies were biomarkers, and the primary endpoint of the safety and efficacy study matched the primary endpoint used in the dose finding study in 9 of 13 (69%) drug development programs where primary study endpoints were assessed.

**Conclusions:**

Our study showed that NME drug development programs for rare genetic diseases utilize multiple data sources for dosing information, including studies in healthy subjects, population pharmacokinetic analyses, and exposure–response analyses. In addition, our results indicate that biomarkers play a key role in dose-finding studies for rare genetic disease drug development programs. Our findings highlight the need to develop study designs and methods to allow adequate dose-finding efforts within rare disease drug development programs that help overcome the challenges presented by low patient prevalence and other factors. Furthermore, the frequent reliance on biomarkers as endpoints for dose-finding studies underscores the importance of biomarker development in rare diseases.

## Background

The development of therapies for rare diseases poses a myriad of challenges, including difficulties enrolling patients into studies, variability in the course of disease, a lack of well-established study endpoints to support drug approval, the perceived lack of economic incentive for drug developers, and many others. In addition, many rare diseases are genetic, and this presents additional challenges because genetic diseases often have genetic and phenotypic heterogeneity [1]. Therefore, the age of onset, disease severity, and prognosis can be highly variable, which creates multiple challenges, including identifying safe and effective dosing regimens and developing endpoints to measure responses in these small patient populations.

The Orphan Drug Act of 1983 and other incentive programs have been introduced to foster and expedite development of orphan drugs, which are defined as therapies used to treat diseases with less than 200,000 individuals in the United States [2–4]. Incentives provided by these programs include marketing exclusivity, tax credits, and access to additional grant programs [3, 5]. In addition, regulations state that it is appropriate for the FDA to exercise the broadest flexibility in applying the regulatory standards for therapies developed to treat rare diseases [6, 7]. As a result of these and other factors, rare disease drug development has rapidly increased in recent years; however, effective therapeutics are still lacking for the vast majority of rare genetic diseases [1, 8].

Drug development and approval for rare diseases should balance drug development challenges and medical need against the need to demonstrate that a therapy's benefits outweigh its risks. While regulations indicate it is appropriate for the FDA to exercise flexibility in applying the regulatory standards for drugs to treat serious and life-threatening diseases, the FDA also must preserve appropriate guarantees for safety and effectiveness [6, 7]. Optimizing the benefit-risk profile of drugs through appropriate dose selection during drug development is critical for all drugs to reduce the likelihood that a truly effective drug will fail to demonstrate efficacy (because the dose studied was too low) or have unacceptable risks (because the dose is too high). In fact, the inability to determine a suitable dose for drug labeling is the most commonly identified reason for non-approval of a drug during its initial review cycle by the FDA [9]. For orphan drugs, dose selection may be even more important because often only one clinical safety and efficacy trial is feasible [6].

Dose optimization relies on a thorough understanding of dose-exposure–response relationships. While data from many sources are necessary to adequately characterize dose-exposure–response relationships, understanding a drug’s pharmacokinetic (PK) and pharmacodynamic (PD) effects over a wide range of dosages is essential [10]. These data are most commonly attained through early phase “dose-finding” studies, where multiple dosage regimens are administered to patients and drug PK and response, including safety and efficacy (PD or clinical outcomes) endpoints, are carefully measured. The information from such studies is then used to inform the dosing strategy for larger safety and efficacy trials, as well as dosage adjustments for specific populations based on intrinsic and extrinsic factors. However, given the limited number of patients available for clinical studies within a rare genetic disease patient population, adequate dose-finding studies are challenging to conduct and may not be performed consistently in these drug development programs [11, 12]. Understanding both the challenges associated with conducting dose-finding studies in rare disease populations and the importance of dose selection and optimization for successful drug development, we sought to characterize the dose-finding studies and analyses conducted for recently approved drugs to provide insight on current practices for dose-finding in rare genetic disease drug development.

## Results

**Fig. 1:**
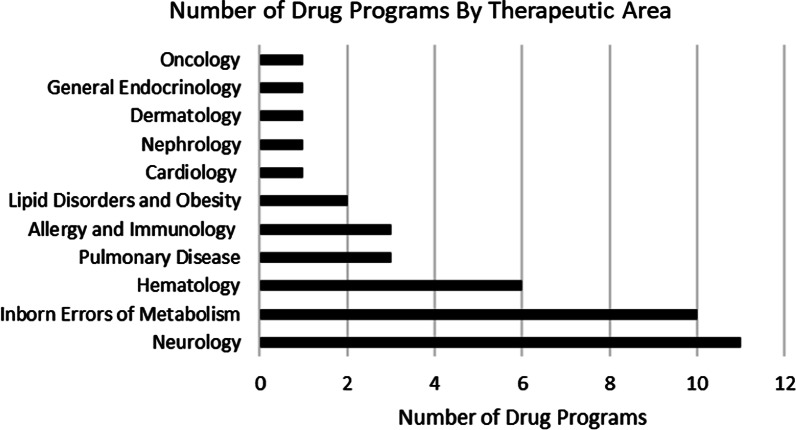
The number of drug development programs analyzed by therapeutic area

Forty new molecular entities (NMEs) were approved for rare genetic diseases between 2015 and 2020. Seventeen of the approvals (43%) were small molecule drugs, 8 (20%) were oligonucleotide therapeutics, 6 (15%) were monoclonal antibodies, 6 (15%) were enzyme-replacement therapies, and 3 (7%) were for other molecule types. The most common indications were for neurologic diseases (n = 11; 28%), inborn errors of metabolism (n = 10; 25%), and hematologic diseases (n = 6; 15%) (Fig. 1). The patient population sizes for the indicated populations varied considerably. On one end of the spectrum, several NMEs were approved for indications with less than 1,000 patients in the U.S., while at the other end of the spectrum, several NMEs were approved for indications with more than 100,000 patients in the U.S. (Table 1). The age of onset for the diseases that the drugs were approved for also varied, but most (36 of 40, 90%) could appear in infants or pediatrics; only 4 of 40 (10%) were present in adults only.

**Table 1:** U.S. patient population prevalence estimates for rare genetic disease drug development programs

Patient population size	Number of programs
< 1000	6
1000 to < 10,000	10
10,000 to < 100,000	18
100, 000 to < 200,000	6

### Dose-finding studies

**Fig. 2:**
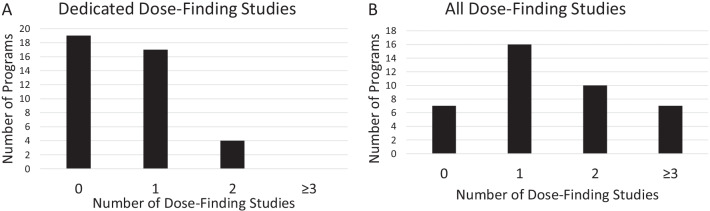
Number of (**A**) dedicated dose-finding studies and (**B**) all dose-finding studies conducted within drug development programs

Of the 40 drug development programs for rare genetic diseases, 21 (53%) conducted at least one dedicated dose finding study (Table 2). Nineteen (48%) drug development programs did not conduct any dedicated dose-finding studies, 17 (43%) conducted one dedicated dose-finding study, 4 (10%) conducted two dedicated dose-finding studies, and none conducted more than two, which resulted in a total of 25 dedicated dose-finding studies across the 40 drug development programs (Fig. 2A). When considering all dose-finding studies (i.e., including titration studies and pivotal trials with more than one dosing arm), 7 (18%) drug development programs did not conduct any dose-finding studies, 16 (40%) conducted one dose-finding study, 10 (25%) conducted two dose-finding studies, and 7 (18%) conducted three or more dose-finding studies (Fig. 2B). There were 14 (35%) drug development programs that conducted both at least one dedicated dose-finding study and at least one additional study that included dose-finding elements.

**Table 2:** Types of dose-finding studies and analyses performed in rare genetic disease drug development programs

Type of study or analysis	All drug development programs (n = 40)	No dedicated dose-finding study (n = 19)
Dedicated dose-finding study	21 (53%)	Not applicable
Healthy subject study	23 (58%)	11 (58%)
Study in different patient populations*	10 (25%)	4 (21%)
Population pharmacokinetic analysis	31 (78%)	13 (68%)
Exposure–response analysis	28 (70%)	11 (58%)

*Different patient population refers to patient populations being studied for a disease other than the rare genetic disease for which the drug was initially approved

**Fig. 3:**
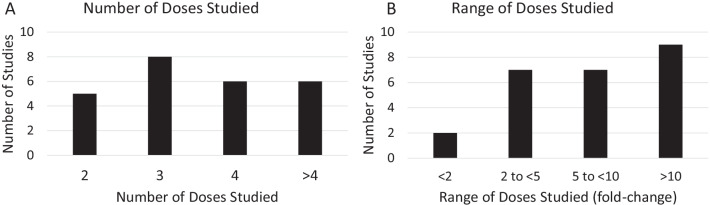
**A** Number and **B** range of doses studied in dedicated dose-finding studies

The number of individual dosage regimens studied in dedicated dose-finding studies was variable, ranging from two to eight doses (Fig. 3A). Similarly, the range of dosage regimens included in dedicated dose-finding studied varied substantially. While some studies included less than a 2-fold range from the highest to the lowest dose studied, more commonly a broader range was assessed (Fig. 3B). In fact, 9 of the 25 dedicated dose-finding studies (36%) had more than a 10-fold range from the highest to lowest dose studied.

### Additional dose-finding data

In addition to dedicated dose finding studies, many drug development programs included other studies or analyses that may be used to inform dose selection and optimize the dosing regimen. The majority of drug development programs conducted clinical studies in healthy subjects, and some had conducted clinical studies in patient populations other than the disease for which the drug was initially approved (Table 2). Moreover, the majority of drug development programs included population PK analyses and included exposure–response analyses in the submission (Table 2).

We further analyzed the 19 drug development programs that did not have a dedicated dose-finding study. Twelve (63%) had a study with at least one dose-finding element (i.e., a study that used a titration regimen or a safety and efficacy trial that included more than one dose). Similar to analysis of all drug development programs, the majority of drug development programs that did not conduct a dedicated dose-finding study conducted studies in healthy subjects and included population PK analyses and exposure–response analyses, while a minority included dose-finding studies from other patient populations (Table 2). Overall, the frequency of these additional studies or analyses was similar when comparing all drug development programs to the programs that did not conduct a dedicated dose-finding study.

### Characterization of endpoints used in dedicated dose-finding trials and confirmatory trials

Thirteen of the dedicated dose-finding studies identified a clinical outcome or PD biomarker as the primary endpoint. The vast majority of primary endpoints utilized in dedicated dose-finding studies were biomarkers, while clinical outcomes or a combination of biomarkers with clinical outcomes or COAs were also used (Table 3). When compared to the primary endpoint of the confirmatory efficacy study, 9 of the 13 (69%) dedicated dose-finding studies had at least one primary endpoint match.

**Table 3:** Categories of primary and secondary endpoints used in dose-finding studies

Category of primary endpoint	Number of dedicated dose-finding studies (n = 13)
Biomarker	10 (77%)
Clinical outcome	1 (8%)
Biomarker, clinical outcome assessment	1 (8%)
Biomarker, clinical outcome	1 (8%)

Category of secondary endpoint	Number of dedicated dose-finding studies (n = 22)
Biomarker	14 (64%)
Clinical outcome	2 (9%)
Biomarker, clinical outcome assessment	4 (18%)
Biomarker, clinical outcome assessment, clinical outcome	1 (5%)
Clinical outcome, clinical outcome assessment	1 (5%)

Twenty-two of the dedicated dose-finding trials listed an efficacy measure as a secondary endpoint. Similar to the analysis of primary endpoints, biomarkers were the most commonly used secondary endpoints, while clinical outcomes and COAs were used more rarely (Table 3). When compared to the primary endpoint of the confirmatory efficacy study, 13 of the 22 (59%) of the secondary endpoints from dedicated dose-finding studies matched the primary endpoint from the confirmatory efficacy study. In total, 18 of the 25 (72%) of the dedicated dose finding studies had a primary and/or secondary endpoint that matched the primary endpoint of the confirmatory efficacy study. Of note 32 of 61 confirmatory trials (52%) used at least one biomarker as a primary endpoint.

## Discussion

Dose-finding studies are a critical component of drug development and failure to identify a safe and effective dose can cause clinical trial failures, and is frequently cited as a reason for non-approval of drugs [9]. Rare genetic diseases present many challenges in conducting robust dose-finding studies; however, dose selection and optimization may be even more important for rare genetic diseases because of the challenges associated with conducting confirmatory efficacy trials in this setting. Therefore, we systematically reviewed the clinical trial information included in recently approved NMEs for rare genetic diseases to characterize dose-finding efforts in these drug development programs. Our results demonstrate that while the majority of drug development programs conducted a dedicated dose-finding study, many did not include these important studies. In addition, our findings indicate that biomarkers play a key role in dose-finding studies for rare genetic diseases and highlight the importance of biomarker development for rare genetic diseases.

In drug development, dose selection for confirmatory efficacy studies is often based on dose-finding studies that assess a wide range of dosages in the relevant patient population and generate robust data on dose-exposure–response relationships. These data are then used to identify the dosing regimen or regimens that are most likely to demonstrate safety and efficacy in later studies, and thus most likely to meet the regulatory standards for marketing authorization. Our study showed that just over half of NME drug development programs for rare genetic diseases conducted a dedicated dose-finding study. Given the challenges associated with conducting clinical studies in rare disease populations, alternative methods to generating dose-exposure–response data to inform dose selection may be appropriate, such as titrating the dose within a study or evaluating multiple dosage regimens in late phase safety and efficacy study. After accounting for all of these methods, a significant number of drug development programs remained (18%) that did not conduct any dose-finding studies. These findings may indicate that alternative strategies are being used to generate data to inform dose selection and optimization for drugs used to treat rare genetic diseases or may suggest that dose finding is not emphasized or prioritized in these programs, which could potentially lead to non-approval of an effective drug that was improperly dosed or approval of a drug at a dosage that does not have an optimal benefit-risk profile. However, one limitation of our analysis is that we did not compare drug development programs that ultimately resulted in an approved product to those that did not; as such, we cannot make a direct conclusion about the impact of conducting these studies on approval or the approved dosing regimens.

We found that the dose-finding studies conducted within NME drug development programs for rare genetic diseases usually included at least 3 and often 4 or more dosage regimens, and the majority included dosage regimens that spanned at least a 5-fold range and often greater than a 10-fold range. The use of several dosages and a wide range of dosages is critical to establishing dose–exposure–response relationships to adequately inform dose selection and optimization [13], as well as the need for dose adjustment based on intrinsic or extrinsic factors (e.g., organ impairment or drug interactions). Although we did not assess all aspects of study conduct and quality, the basic design of these dose-findings studies appears adequate to obtain the necessary data for adequate dose finding. Appropriate design and conduct of dose-finding studies is crucial not only for dose optimization and dose selection, but also because exposure–response information can add to the weight of evidence of drug effectiveness and an acceptable benefit-risk profile that support approval [14]. This evidence may be critical in the setting of rare diseases, where it may not be ethical or feasible to conduct more than one adequate and well-controlled clinical investigation, and thus confirmatory evidence such as biomarker data and exposure–response information may need to be relied upon to support approval of the drug [15].

Our analysis focused on dose-findings studies as a principal data source for dose selection and optimization. However, other methods may also provide valuable data regarding dose–exposure–response relationships that can inform dose and optimization; therefore, we also assessed the frequency that data from healthy subject studies, other patient populations, and data from population PK and exposure–response analyses were submitted in support of the application. We found that the majority of NME drug development programs conducted clinical studies in healthy subjects and included population PK analyses and exposure–response analyses, while only a few programs also included dose-finding data from other patient populations in their submissions. The use of these alternative data sources did not appear more common in programs that did not conduct a dedicated dose-finding study, so we cannot conclude that these methods are relied upon when dedicated studies are not feasible; however, they do appear to be used frequently to provide information within rare genetic disease drug development programs. These, and other innovative methods of characterizing dose–exposure–response information, such as microdosing studies, model-informed drug development approaches or complex innovative trial designs, that rely on quantitative models derived from preclinical and clinical data sources, can be used to provide valuable information on dose selection and optimization and help increase the probability of regulatory success in the absence of dedicated dose-finding trials [16–19].

Biomarkers are used for many purposes in drug development, particularly as measures of drug response in dose-finding studies. We found that biomarkers were by far the most commonly used primary and secondary endpoints for dedicated dose-finding studies, and the same biomarkers were frequently the primary endpoint in the confirmatory efficacy trial. The use of biomarkers for endpoints in dose finding studies is reasonable, and may be preferred over clinical outcomes because biomarkers are often more sensitive to drug effects and more directly related to drug plasma concentrations compared to measuring clinical outcomes, which can be impacted by multiple factors [14]. In addition, biomarkers often allow collection of information on drug effect in smaller numbers of patients in a shorter duration of treatment compared to clinical outcomes, thus minimizing the amount of time a patient participating in a study would receive a dose that is unlikely to be effective, which is particularly important in rare genetic diseases which are often chronic and progressive. However, the relationship between a change in a biomarker and the clinical outcome of interest often is not adequately characterized. The repeat assessment of disease-related PD biomarkers used as endpoints in dose-finding studies in the subsequent confirmatory efficacy trials can provide confirmatory evidence of pharmacologic activity on the causal path, thus linking the PD effect to the clinical outcome [20].

Biomarkers may be able to help address some of the major challenges in rare disease drug development, such as practical dose-finding studies that inform dose selection and optimization, generating confirmatory evidence of effectiveness (to be used along with a clinical outcome endpoint to help support approval), and ultimately as surrogate endpoints for approval in lieu of measuring clinical outcomes. However, efforts are needed to appropriately develop and validate biomarkers for these purposes. To accomplish this, a stepwise approach could be used where a thorough understanding of the mechanistic role of the biomarker in the disease, coupled with data that correlates the biomarker to clinical outcomes, could initially support use of the biomarker as an endpoint for dose-finding studies before data are generated to support the use of the biomarker as a validated surrogate endpoint in efficacy trials. Although this approach involves some risk because of the uncertainty of the biomarker’s ability to predict the clinical outcome prior to being validated as a surrogate endpoint, this may be an acceptable trade-off to allow informative dose finding studies that are more practical to conduct.

## Conclusions

Our study showed that NME drug development programs for rare genetic diseases utilize several different data sources for information on dosing. However, a significant number of drug development programs did not have any clinical studies classified as a dose-finding study. In addition, we showed that biomarkers play a key role in dose-finding studies for rare genetic disease drug development programs. Our findings highlight the need to develop study designs and methods to allow adequate dose-finding efforts within rare disease drug development programs that help overcome the challenges presented by low patient prevalence, variable course of disease, and other factors. Furthermore, the frequent reliance on biomarkers as endpoints for dose-finding studies underscores the importance of biomarker development in rare diseases.

## Methods

### Identification of rare genetic disease products

Rare genetic diseases were defined as diseases caused by germline genetic alterations and meeting the U.S. criteria for a rare disease (prevalence of < 200,000 patients in United States) [4]. To identify drug development programs for recently approved NMEs (inclusive of small molecule drugs and biological products) for rare genetic diseases, we first identified all NMEs approved from 2015–2020 using the FDA’s Data Analysis Search Host (DASH) database. DASH is an internal FDA database that collects regulatory properties of drug development programs for NMEs. Disease prevalence was estimated by the FDA’s Office of Orphan Products Development at the time each request for orphan drug designation was granted; since the purpose of orphan drug designation is to determine whether the affected population is below 200,000, these estimates often rely on the largest reasonable prevalence estimate and are not considered the official prevalence for each disease or condition. NMEs for rare diseases were identified using DASH, and of those, NME applications for rare genetic diseases were identified and confirmed by two authors. Therapeutic area categorizations were made independently by two authors and differences were resolved by discussion.

### Identification and assessment of dose-finding studies

Dedicated dose-finding studies were defined as phase 1 or 2 clinical studies in the disease population for which the drug was initially approved that studied more than one dosing regimen in parallel and included an efficacy measure as a primary or secondary objective or endpoint. Studies were not counted as dedicated dose-finding studies if only a single dose was administered (for a chronically administered drug), the study was an extension of a previous study, or the study was the phase 3 confirmatory efficacy study for the drug.

All clinical studies conducted as part of individual drug development programs were identified from the Table of Clinical Studies submitted with the application. In addition to dedicated dose-finding studies, study information was collected from clinical studies that utilized titration and confirmatory clinical trials that included more than one dosing arm because these studies contain some features of dose-finding studies (e.g., study of more than one dosage, collection of response information) and data from these studies can also be used to inform dose selection and optimization; the combination of dedicated dose-finding studies and additional studies that incorporate these features are referred to as “all dose-finding studies” within this manuscript. For each dose-finding study, the number of dosage regimens and range of doses evaluated were captured from the study report.

We also determined whether sponsors used additional methods (other than dose-finding studies) to inform dose selection and optimization, such as conducting pharmacokinetic studies in healthy subjects or in other patient populations. Healthy subject studies were defined as studies in which multiple dosing arms were studied in healthy subjects. Dose-finding trials in other patient populations were defined as phase 1 or 2 clinical studies that studied multiple dosing arms where an efficacy measure was a primary or secondary objective in patients with a disease other than the one indicated in the initial approval. In addition, we assessed whether sponsors conducted dose-finding analyses other than clinical studies, including exposure–response or population PK analyses, by searching the clinical pharmacology review, which was obtained from Drugs@FDA [21]. We further assessed these additional methods by conducting a subset analyses of programs with no dedicated dose-finding studies to determine if these programs utilized the additional methods more frequently.

### Identification and characterization of endpoints used in dose-finding studies

The primary and secondary endpoints used in dedicated dose-finding studies were collected from the clinical study reports. Endpoints were then categorized as biomarkers, clinical outcomes, or clinical outcome assessments (COAs). Endpoints for confirmatory safety and efficacy trials, defined as the confirmatory efficacy trials cited in the Clinical Studies section (Section 14) of the FDA-approved product labeling (i.e., US Prescribing Information), were identified and cross-checked with the clinical study report submitted by the applicant. To evaluate the continuity of clinical trial endpoints across studies conducted during drug development, we determined if a primary or secondary endpoint for the dedicated dose-finding study was also used as the primary endpoint for the confirmatory efficacy trial(s). To be considered “matched,” the endpoints being compared needed to study the same variable (e.g., LDL cholesterol), but could differ in measurement or analysis aspects (e.g., percent change vs absolute change). For example, if a drug program had a dose-finding study using percent change in bodyweight from baseline as a secondary endpoint and one of the confirmatory trials studied absolute change in bodyweight from baseline as the primary endpoint, these would be considered “matching” endpoints. If a drug development program used composite or co-primary endpoint(s), the endpoints would be considered “matched” if any single endpoint that made up the composite or co-primary endpoint in the confirmatory trial was the same as an endpoint studied in the dose-finding trial.

## Data Availability

The datasets generated and/or analyzed during the current study are not publicly available due to the confidential nature of data submitted to the FDA by third parties; to the extent possible, data will be made available by the corresponding author on reasonable request.
